# Trans-apical aortic valve implantation for quadricuspid aortic valve with aortic regurgitation using J-valve system: a case reports

**DOI:** 10.1186/s13019-021-01586-9

**Published:** 2021-08-03

**Authors:** Chaodi Luo, Yi Jiang, Qiang Chen, Yang Yan, Dan Han

**Affiliations:** 1 Department of Cardiology, the First Affiliated Hospital of Xi’an Jiaotong University, 277 Yanta West Road, Xi’an, Shaanxi 710061 People’s Republic of China; 2 Department of Cardiovascular Surgery, the First Affiliated Hospital of Xi’an Jiaotong University, Xi’an, People’s Republic of China

**Keywords:** Quadricuspid aortic valve, Transapical aortic valve implantation, J-valve

## Abstract

**Background:**

Quadricuspid aortic valve (QAV) is a rare congenital heart defect usually accompanied with different hemodynamic abnormalities. Due to the rarity of QAV, treatment and prognosis of QAV patients with aortic regurgitation still remain challenging. We here present the first case of a patient with severe QAV regurgitation who underwent successful treatment and performed favorable prognosis with transapical aortic valve implantation (TAVI) using J-Valve system.

**Case presentation:**

A 62-year-old man experienced intermittent palpitation, shortness of breath and chest pain. Echocardiography revealed congenital QAV with massive aortic regurgitation and mild aortic stenosis, left ventricular enlargement. Aortic valve replacement was successfully performed with TAVI using J-Valve system. The postoperation and follow-up was uneventful.

**Conclusion:**

TAVI using J-Valve system has emerged as a new high success rate method for treatment of patients with simple non-calcified aortic valve insufficiency.

**Supplementary Information:**

The online version contains supplementary material available at 10.1186/s13019-021-01586-9.

## Introduction

Quadricuspid aortic valve (QAV) is a rare congenital heart defect with an estimated incidence between 0.003 and 0.013%. Patients with QAV usually present with hemodynamic abnormalities, most commonly aortic regurgitation [1]. Diagnosis of QAV based on echocardiography is not difficult, however, the repaired prosthesis durability and long-term outcomes of such non-tricuspid aortic valves remain uncertain [2]. Surgical aortic replacement has long been the first choice for aortic regurgitation, however, it may increase the complications and mortalities for high-risk patients [3]. On the other hand, for patients with simple non-calcified aortic valve insufficiency, many transcatheter aortic valve products are not reliable enough to anchor, and postoperative perivalvular leakage or valve displacement is easy to occur, which limit their applicability [4]. Transapical aortic valve implantation (TAVI) using J-Valve system has emerged as a new method for treatment of high-risk patients with simple non-calcified aortic valve insufficiency [5]. Due to the rarity of QAV, only a few cases of stenosed QAVs treated with transcatheter aortic valve implantation have been reported [6, 7]. We here present the first case of a patient with severe QAV regurgitation who underwent successful treatment and performed favorable prognosis with TAVI using J-Valve system.

## Case presentation

**Fig. 1:**
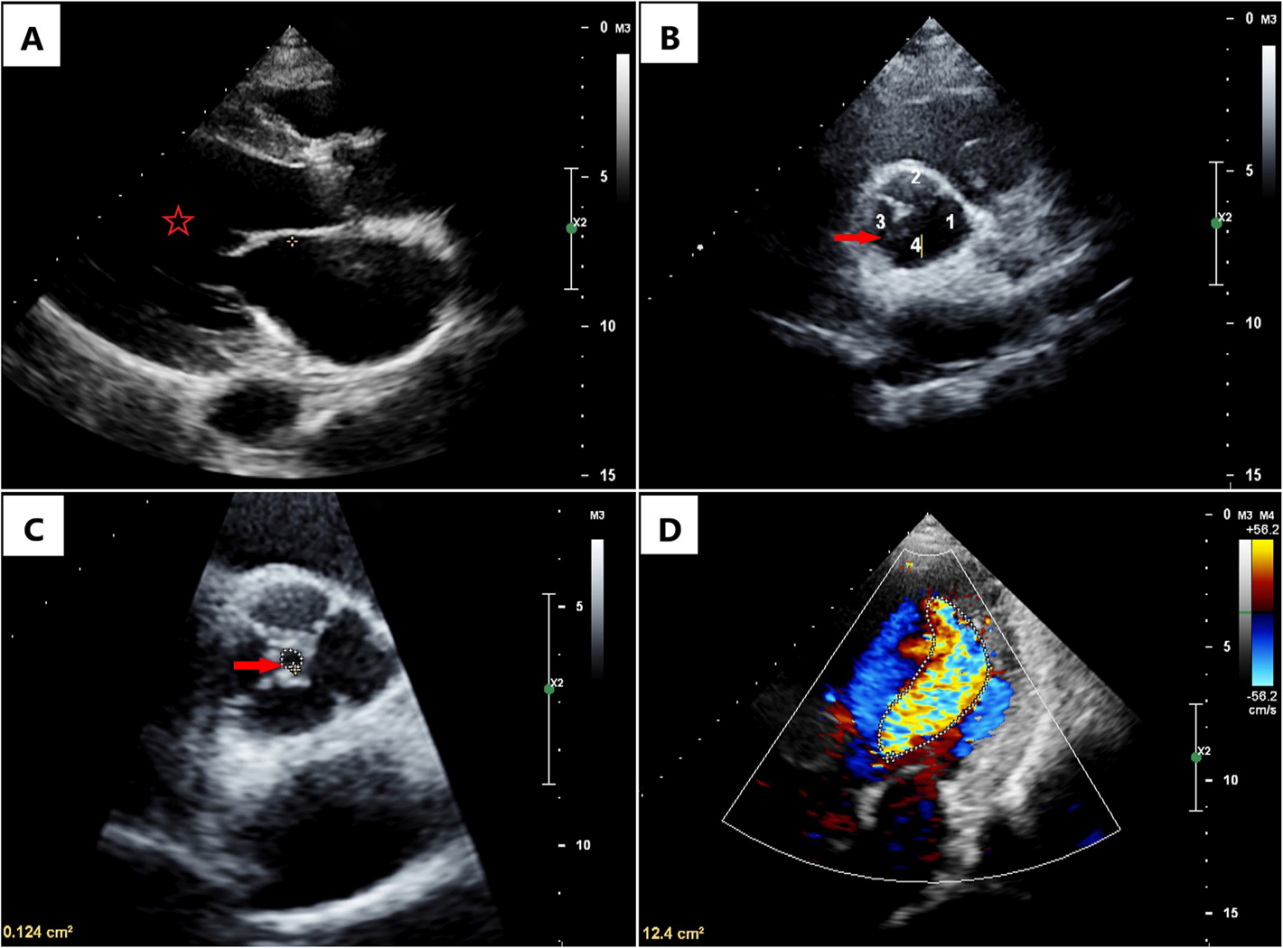
Echocardiography showed left ventricular enlargement (Panel A, red star indicated dilated ventricle), quadricuspid aortic valve (Panel B, red arrow indicated four aortic valves, which have been marked numerically), mild aortic stenosis (Panel C, red arrow indicated the hole formed by aortic insufficiency, the area of which is about 0.124cm^2^) and massive aortic regurgitation (Panel D) of the QAV patient


A 62-year-old man was admitted to our department with a history of hypertension, intermittent palpitation, shortness of breath and chest pain for 10 years. Laboratory examination showed that NT-ProBNP was of 1050 ng/L. Echocardiography revealed congenital QAV (Supplemental video 1) with massive aortic regurgitation (the regurgitant jet area was measured as 12.4 cm^2^) and mild aortic stenosis, left ventricular enlargement (Fig. 1). Ventricular function was normal with the left ventricular ejection fraction of 59%. Cardiac computed tomography (CCT) revealed type A QAV without significant valvular thickening or calcification, average aortic annulus diameter was 25.9 mm, ascending aortic diameter was 31.8 mm, sinotubular junction was 28.8 mm, average left ventricular outflow tract diameter was 28.2 mm. The patient had relatively low coronary ostial height with the left coronary artery arising only 11.5 mm from the valve annulus, and the right coronary ostial height was 14.7 mm (Supplemental Figure 1).

**Additional file 2: Supplemental video 1.** TEE revealed congenital type A QAV.

The patient was prescribed with digoxin 0.125 mg, metoprolol 12.5 mg, spironolactone 20 mg, hydrochlorothiazide 25 mg, and potassium chloride 1 g, all every 8 h after admission to improve his symptoms. After one week of treatment, the above symptoms alleviated significantly and the patient had a strong wish to repair the aortic valve. The patient was evaluated as stage D and the EuroSCORE was 2 [8]. However, due to the patient’s strong refusal of surgical intervention, and the commercially available transcatheter valve implantation was also inappropriate, our heart team recommended TAVI using J-Valve system. The informed consent was obtained from the patient. Hospital ethics committee approval was also granted.

**Fig. 2:**
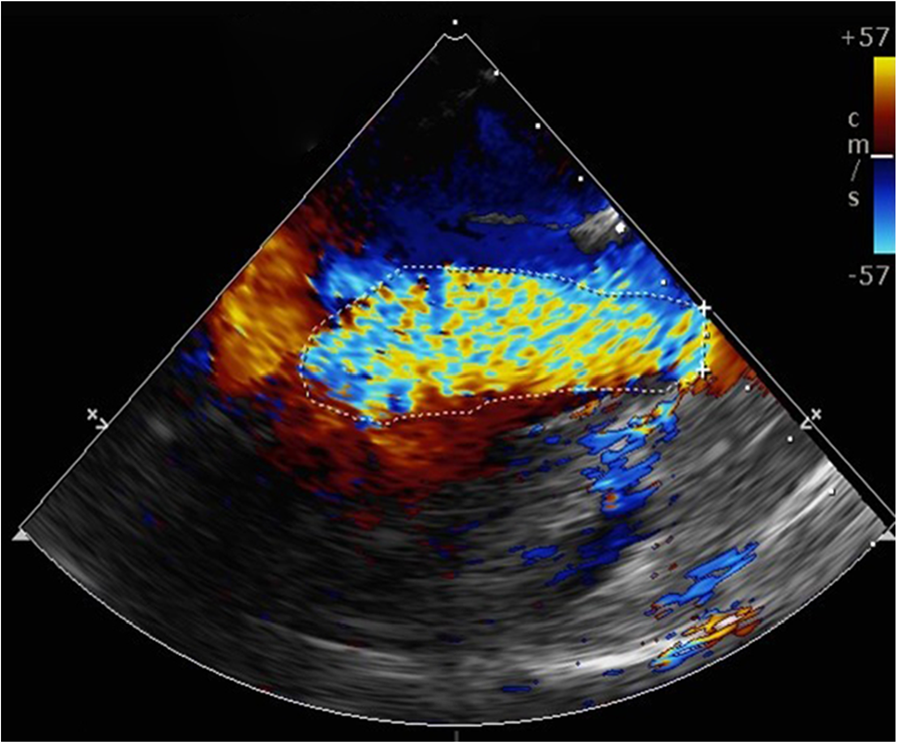
Long axial section of left ventricle of TEE showed massive aortic regurgitation before the procedure

**Fig. 3:**
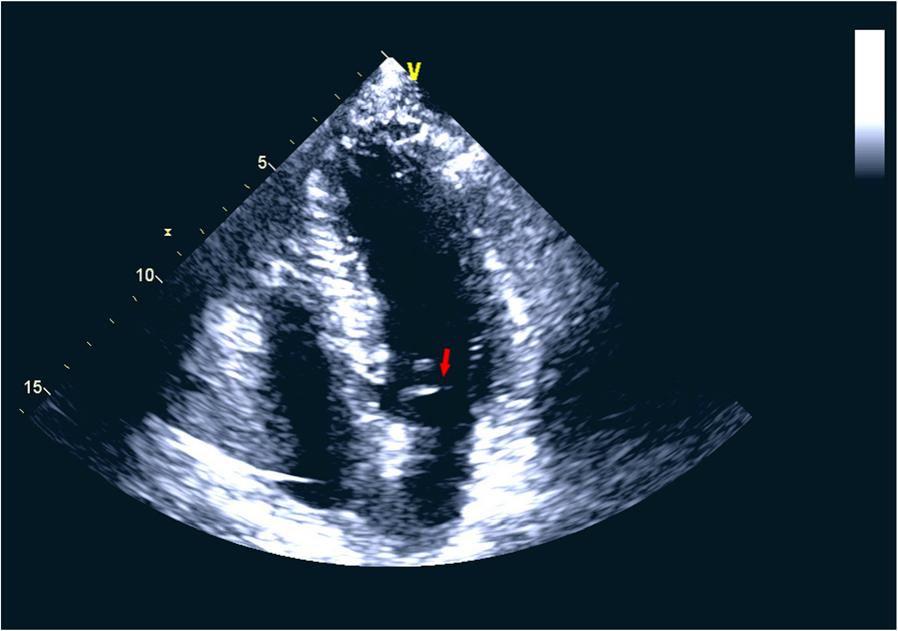
Echocardiography revealed that the artificial aortic valve worked well (red arrow)


Under general anaesthesia, the patient underwent successful implantation of a 27-mm J-Valve via transapical access in a hybrid operating room. At the beginning, a 6F pigtail catheter was inserted into the aortic sinus via the left femoral artery and a temporary pacemaker was implanted to the right ventricular apex via the left femoral vein (Supplemental video 2). Aortic root angiography and transesophageal echocardiography (TEE) were utilized for evaluation of the valve pathology (Fig. 2) and apical position. The C-arm position was adjusted according to the previous CCT data so that the three aortic sinuses are simultaneously displayed and located in the same plane. Next, a 4-cm tiny incision was made at the fifth costal margin to expose the pericardium. The apical puncture was performed, TEE and fluoroscopy showed that super-stiff guidewire was placed in position and did not affect mitral valve tendinous cord (Supplemental video 3). According to preoperative assessment, a 27-mm J-Valve (Jie-cheng Medical Technology, Suzhou, China) was crimped into the delivery system and transported into a supra-annular position following fluoroscopic guidance. With the assistance of pigtail catheter under fluoroscopy and TEE, the three “U-shape” graspers were carefully folded as three “long elliptical shape” in favor of positioning and then totally released into the left-, right- and non-coronary sinuses to clamp the native leaflets (Supplemental video 4). The correct position was confirmed by fluoroscopy and TEE, the valve was then delivered into the annular plan guided by the graspers and released with rapid ventricular pacing at 120 bpm (Supplemental video 5). After withdrawing the delivery system, TEE and aortic root angiography showed no aortic regurgitation as well as no paravalvular leak with patent coronaries and good valve stent position, and the artificial aortic valve worked well (Supplemental videos 6 and 7). There was no intraoperative or perioperative complication. The patient did well postoperatively and the NT-ProBNP was of 1940 ng/L on postoperative recheck. Then he discharged home in good clinical condition after 10 days. Six months later, our patient was followed up at the outpatient, he made a successful recovery, the NT-ProBNP was down to 104 ng/L and enchocardiography revealed that the artificial aortic valve functioned normally, the left ventricular diameter and ventricular function were normal with the left ventricular ejection fraction of 64% (Fig. 3).

**Additional file 5: Supplemental video 4.** The fluoroscopy showed that the three “U-shape” graspers were carefully folded as three “long elliptical shape” in favor of positioning and then totally released into the left-, right- and non-coronary sinuses to clamp the native leaflets.


**Additional file 8: Supplemental video 7.** TEE showed no aortic regurgitation and no paravalvular leak from the view of long axis.

## Discussion

QAV accompanied by different hemodynamic abnormalities is usually treated with surgical valve repair or valve replacement with a synthetic valve. However, surgery may increase the incidence of valve thrombosis, prosthetic valve endocarditis, reduced valve durability, kidney injury, and bleeding complications [3]. Transcatheter valve implantation is now increasingly applied in clinical practice, which includes valve-in-valve treatment for failing bio-prostheses, low-risk patients, native pure aortic regurgitation, and for treatment of congenital valve disease such as bicuspid aortic valves with complex anatomical characteristics [9]. Nevertheless, due to the scarcity of QAV, the durability of prosthetic valve and long-term outcomes remain uncertain. To date, only a few cases of stenosed QAV treated successfully with transcatheter valve implantation have been reported. Unlike aortic stenosis, transcatheter valve implantation is not yet recommended in guidelines for treating pure aortic regurgitation. Some previous off-label clinical experiences showed that the rate of all-cause mortality using the first-generation transcatheter devices in treating pure aortic regurgitation at 30 days was up to 30%, as well as the high incidence of postoperative complications encompassing perivalvular leakage, residual aortic regurgitation, fatal bleeding, major vascular damage, permanent pacemaker implantation, acute kidney injury, and stroke [10, 11]. Therefore, treatment and prognosis of QAV patients with aortic regurgitation are still challenging. In our case, the patient was classified as stage D according to the 2020 ACC/AHA guideline for the management of patients with valvular heart disease [8]. Surgical aortic valve repair was recommended, however, the patient strongly resisted open surgery for valve repair. In addition, CCT showed low coronary artery height with the left coronary artery loading only 11.5 mm from the valve annulus and the right coronary height was 14.7 mm. Using commercially available transcatheter aortic valve replacement would be easily blocking the coronary sinus. Therefore, TAVI using J-Valve would be the most suitable option for this patient.

J-Valve with three “U-shape” graspers is a second-generation self-expandable device which has been approved for treating both aortic stenosis and aortic regurgitation in China. The unique structures of J-Valve system are effective for positioning, anchoring and protecting coronary arteries: (1) the three U-shape graspers are conductive to anchor the leaflets, which decreases the risk of perivalvular leakage and valve displacement; (2) the short path from the apex to the aortic annulus is helpful for adjusting the coaxiality and reducing major vascular damage; (3) because the fixation of J-Valve does not need a robust radial support force, it can be released at a lower level to reduce the rate of conduction block; (4) the low profile and bare metal area are designed for graspers to avoid coronary occlusion, especially for further coronary recanalization after primary valve replacement [12].

Liu et al. [13] reported that a success rate of 97.7% and a mortality rate of 4.7% were observed in patients with pure aortic regurgitation treated by J-Valve. The rate of permanent pacemaker implantation was 4.7, 2.3% patients suffered stroke, and the treatment for failing prostheses rate was 7.0%. During 1-year follow-up, only one patient had mild perivalvular leakage. Xue et al. [12] discovered that the success rate of J-Valve implantation was 91.3%, and the mortality was 4.3%. No cases underwent permanent pacemaker implantation and only one patient suffered mild stroke. No paravalvular leakage was observed during the follow-up. To sum up, the new-generation devices such as J-Valve had a higher device success and a significant reduction in postoperative complications compared to the old-generation.

It is worth noting that the angles among the adjacent graspers are 120°, whereas the angles of adjacent QAV leaflets are 90°. Considering the mismatching between the graspers and the leaflets, we carefully rotated the graspers during the procedure to ensure them capturing the leaflets instead of translocation. Second, valve positioning was fluoroscopically challenging. We folded the three “U-shape” graspers as three “long elliptical shape” under fluoroscopy, which was very helpful for positioning. Third, the position of the left main coronary ostia was relatively low, which needed to be prudently considered in case of coronary occlusion.

## Conclusion

In conclusion, our case adds to the body of evidence supporting the fact that TAVI using J-Valve in treating QAV patient with severe aortic regurgitation is feasible and showed favorable prognosis. Valve positioning and low left main coronary ostia should be prudently considered during the procedure.

## Supplementary Information

**Additional file 1: Supplemental Fig. 1.** Cardiac computed tomography (CCT) revealed type A QAV without significant valvular thickening or calcification, average aortic annulus diameter was 25.9 mm (Panel A), average left ventricular outflow tract diameter was 28.2 mm (Panel B), sinus of valsalva diameters were 33.1 mm and 31.8 mm (Panel C), sinotubular junction was 28.8 mm (Panel D), ascending aortic diameter was 31.8 mm (Panel E), the left coronary ostial height was 11.5 mm (Panel F), the right coronary ostial height was 14.7 mm (Panel G), and the angle between the left ventricular outflow tract and apex was 147° (Panel H).

**Additional file 3: Supplemental video 2.** The fluoroscopy showed a pigtail catheter was inserted into the aortic sinus and a temporary pacemaker was implanted to the right ventricular apex.

**Additional file 4: Supplemental video 3.** The fluoroscopy showed that super-stiff guidewire was placed into the abdominal aorta and did not affect mitral valve tendinous cord.

**Additional file 6: Supplemental video 5.** The fluoroscopy showed that the valve was delivered into the annular plan guided by the graspers and released.

**Additional file 7: Supplemental video 6.** The aortic root angiography showed a good valve stent position, and the artificial aortic valve worked well with patent coronaries.

## Data Availability

The datasets of the current study are available from the corresponding author upon reasonable request.

## Abbreviations

QAV: Quadricuspid aortic valve
TAVI: transapical aortic valve implantation
CCT: Cardiac computed tomography
TEE: Transesophageal echocardiography

## References

[1] Yuan SM (2016). Quadricuspid aortic valve: a comprehensive review. Braz J Cardiovasc Surg.

[2] Mastrobuoni S, Aphram G, Tamer S, Navarra E, de Kerchove L, el Khoury G (2019). Quadricuspid aortic valve repair. Ann Cardiothoracic Surg.

[3] Lorca R, Álvarez-Cabo R, Calvo J, de la Hera JM (2018). Quadricuspid aortic valve surgical repair. J Thorac Cardiovasc Surg.

[4] Howard C, Jullian L, Joshi M (2019). TAVI and the future of aortic valve replacement. J Card Surg.

[5] Ye J, Lee AJ, Blanke P, Webb J (2018). The first transapical transcatheter aortic valve-in-valve implantation using the J-valve system into a failed biophysio aortic prosthesis in a patient with high risk of coronary obstruction. Catheterization Cardiovasc Interventions.

[6] Benkemoun H, Bramlage P (2020). A four-leaf clover: A case report of quadricuspid aortic valve stenosis. Card Surg.

[7] Ibrahim M, Wattanakit K, Barzallo M, Mungee S (2018). Quadricuspid aortic valve stenosis: expanding our experience in Transcatheter aortic valve implantation. J Invasive Cardiology.

[8] Otto CM, Nishimura RA, Bonow RO, Carabello BA, Erwin JP, Gentile F, Jneid H, Krieger EV, Mack M, McLeod C, O'Gara PT, Rigolin VH, Sundt TM, Thompson A, Toly C (2021). 2020 ACC/AHA guideline for the Management of Patients with Valvular Heart Disease: a report of the American College of Cardiology/American Heart Association joint committee on clinical practice guidelines. Circulation..

[9] Puri R, Iung B, Cohen DJ, Rodés-Cabau J (2016). TAVI or no TAVI: identifying patients unlikely to benefit from transcatheter aortic valve implantation. Eur Heart J.

[10] Franzone A, Piccolo R, Siontis GC (2016). Transcatheter aortic valve replacement for the treatment of pure native aortic valve regurgitation: a systematic review. JACC Cardiovasc Interventions.

[11] Yoon SH, Schmidt T, Bleiziffer S, Schofer N, Fiorina C, Munoz-Garcia AJ, Yzeiraj E, Amat-Santos IJ, Tchetche D, Jung C, Fujita B, Mangieri A, Deutsch MA, Ubben T, Deuschl F, Kuwata S, de Biase C, Williams T, Dhoble A, Kim WK, Ferrari E, Barbanti M, Vollema EM, Miceli A, Giannini C, Attizzani GF, Kong WKF, Gutierrez-Ibanes E, Jimenez Diaz VA, Wijeysundera HC, Kaneko H, Chakravarty T, Makar M, Sievert H, Hengstenberg C, Prendergast BD, Vincent F, Abdel-Wahab M, Nombela-Franco L, Silaschi M, Tarantini G, Butter C, Ensminger SM, Hildick-Smith D, Petronio AS, Yin WH, de Marco F, Testa L, van Mieghem NM, Whisenant BK, Kuck KH, Colombo A, Kar S, Moris C, Delgado V, Maisano F, Nietlispach F, Mack MJ, Schofer J, Schaefer U, Bax JJ, Frerker C, Latib A, Makkar RR (2017). Transcatheter aortic valve replacement in pure native aortic valve regurgitation. J Am Coll Cardiol.

[12] Xue Y, Zhou Q, Li S, Li J, Mu D, Luo X, et al. Trans-apical transcatheter valve replacement using J-valve for aortic valve diseases. Ann Thorac Surg. 2020. 10.1016/j.athoracsur.2020.10.030. 10.1016/j.athoracsur.2020.10.030 33248996

[13] Liu H, Yang Y, Wang W, Zhu D, Wei L, Guo K, Zhao W, Yang X, Zhu L, Guo Y, Wang W, Wang C (2018). Transapical transcatheter aortic valve replacement for aortic regurgitation with a second-generation heart valve. J Thorac Cardiovasc Surg.

