# Supplementary figures and images for: Trans-apical aortic valve implantation for quadricuspid aortic valve with aortic regurgitation using J-valve system: a case reports
Source: J Cardiothorac Surg. 2021 Aug 3;16:215. doi: 10.1186/s13019-021-01586-9 (PMC8330120; doi:10.1186/s13019-021-01586-9)

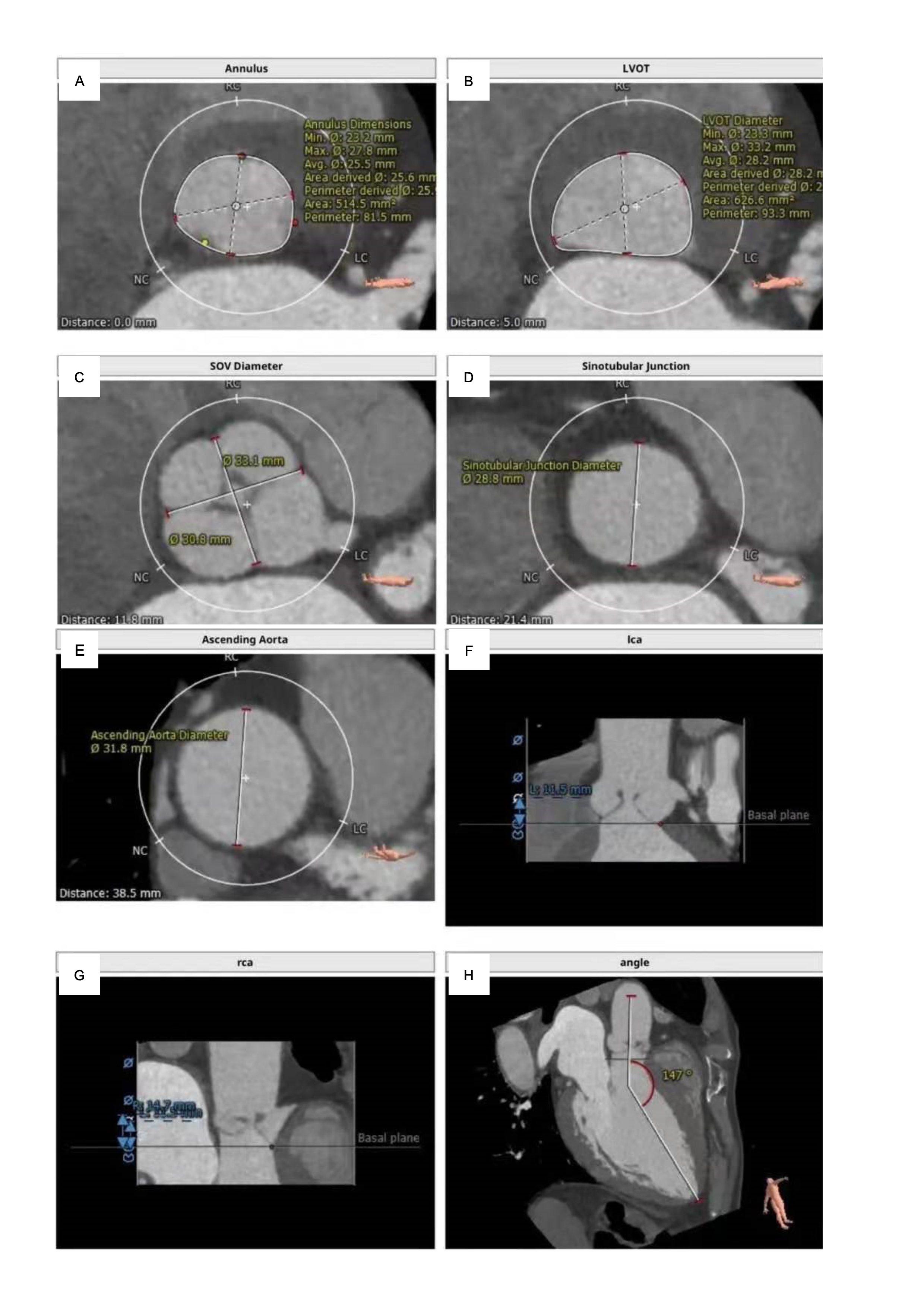

Supplement: Supplementary file 4 — Additional file 1: Supplemental Fig. 1. Cardiac computed tomography (CCT) revealed type A QAV without significant valvular thickening or calcification, average aortic annulus diameter was 25.9 mm (Panel A), average left ventricular outflow tract diameter was 28.2 mm (Panel B), sinus of valsalva diameters were 33.1 mm and 31.8 mm (Panel C), sinotubular junction was 28.8 mm (Panel D), ascending aortic diameter was 31.8 mm (Panel E), the left coronary ostial height was 11.5 mm (Panel F), the right coronary ostial height was 14.7 mm (Panel G), and the angle between the left ventricular outflow tract and apex was 147° (Panel H). [file 13019_2021_1586_MOESM1_ESM.tif]
